# A Performance Improvement Strategy for Concrete Damage Detection Using Stacking Ensemble Learning of Multiple Semantic Segmentation Networks

**DOI:** 10.3390/s22093341

**Published:** 2022-04-27

**Authors:** Shengyuan Li, Xuefeng Zhao

**Affiliations:** 1 School of Mechanics and Civil Engineering, China University of Mining and Technology, Xuzhou 221116, China; 2 State Key Laboratory of Coastal and Offshore, Engineering School of Civil Engineering, Dalian University of Technology, Dalian 116024, China; zhaoxf@dlut.edu.cn

**Keywords:** concrete damage detection, semantic segmentation networks, stacking ensemble learning, softmax regression

## Abstract

Semantic segmentation network-based methods can detect concrete damage at the pixel level. However, the performance of a single semantic segmentation network is often limited. To improve the concrete damage detection performance of a semantic segmentation network, a stacking ensemble learning-based concrete crack detection method using multiple semantic segmentation networks is proposed. To realize this method, a database including 500 images and their labels with concrete crack and spalling is built and divided into training and testing sets. At first, the training and prediction of five semantic segmentation networks (FCN-8s, SegNet, U-Net, PSPNet and DeepLabv3+) are respectively implemented on the built training set according to a five-fold cross-validation principle, where 80% of the training images are used in the training process, and 20% training images are reserved. Then, in predicting the results of reserved training images from trained semantic segmentation networks, the class labels of all pixels are collected, and then four softmax regression-based ensemble learning models are trained using the collected class labels and their true classification labels. The trained ensemble learning models are applied to regressed testing results of semantic segmentation network models. Compared with the best single semantic segmentation network, the best ensemble learning model provides performance improvement of 0.21% PA, 0.54% MPA, 3.66% MIoU, and 0.12% FWIoU, respectively. The study results show that the stacking ensemble learning strategy can indeed improve concrete damage detection performance through ensemble learning of multiple semantic segmentation networks.

## 1. Introduction

Concrete structures are usually subject to erosion through wind, earthquakes, fatigue, human factors, and the degradation of the material performance. As a result, superficial damages are produced. The damages intuitively indicate the condition and serviceability of a concrete structure. Cracking and spalling are the most common types of concrete damage. Thus, it is important to detect the superficial cracking and spalling of concrete structures accurately. However, current visual inspection methods, which are conducted manually by experienced inspectors, are extremely time-consuming and labor-intensive [1]. Moreover, the accuracy of damage detection is likely to be influenced by the skill level and experience of the inspectors.

To tackle these limitations, image processing techniques have been widely explored to detect structural damages, such as concrete cracks [2,3,4], spalling [5], potholes and cracks in asphalt pavement [6,7,8]. Histogram or threshold-based morphological approaches can recognize concrete damage from images [9,10]. Based on the recognition result, classification and quantification of the recognized damage are carried out according to some assumptions, such as color, texture, and shape of damage [11]. To increase performance of image processing techniques, general global transforms, and local edge detection of damage images are introduced into damage detection, such as fast Haar transform, fast Fourier transform, Sobel and Canny edge detectors. For transforms and detectors, Abdel-Qader et al. proposed that the fast Haar transform is relatively more reliable for crack detection, and its accuracy is potentially improvable [12]. Based on this, many researchers carried out relevant research and put forward several improvement routes of image processing techniques [13,14,15,16]. However, the results of these previous studies have demonstrated that each image processing technique can only recognize and detect specific structural damage. In addition, the robustness of image processing techniques leaves much to be desired because noises in the images of damage substantially affect detection performance [17].

To overcome the shortcomings of image processing techniques, machine learning is a feasible approach for the detection of concrete damage from images [18,19]. Traditional machine learning-based concrete damage detection approaches apply artificial neural network [20,21], support vector machine [22,23], and Boltzmann machine [24]. Most of the aforementioned approaches generally rely on manually pre-extracted low-level damage features, so they still have limitations in real-world situations owing to their manual feature extraction approach.

Unlike traditional machine learning, deep learning can automatically extract high-level damage features from original images [25]. Based on this, deep learning provides an end-to-end route for automatic damage detection. Convolution neural networks have a strong ability to classify images duo to learning depth features directly from training data. Convolution neural network-based methods greatly improve concrete damage detection technology [26,27,28,29]. However, the convolution neural network-based concrete damage detection methods have to locate and classify damage using an exhaustive search strategy with a sliding window. An object detection network is a feasible solution to locate and classify objects from images automatically, and the object detection network-based methods greatly reduce the time needed for damage detection [30,31,32,33]. However, the detected damage in bounding boxes by the object detection network includes background pixels, which leads to difficulty in quantifying the detected damage. Semantic segmentation networks can locate and classify concrete damage at the pixel level [34,35]. Following the detected damage by semantic segmentation network-based methods, the quantification of detected damage can be implemented by a series of image post-processing techniques [36,37]. However, the damage detection performance of a single semantic segmentation network is generally limited by network architecture and training data.

To improve the detection performance of single semantic segmentation network, this paper proposed a stacking ensemble learning-based concrete damage detection strategy using FCN-8s [38], SegNet [39], U-Net [40], PSPNet [41], and DeepLabv3+ [42]. The above semantic segmentation network can finish the damage detection task individually [43,44]. The proposed method can detect cracking and spalling at the pixel level, which inherits the advantage of a single semantic segmentation network. Furthermore, it should be emphasized that the stacking ensemble learning strategy can improve damage detection performance through regressing damage prediction results of semantic segmentation networks.

The remainder of this paper is organized as follows. Section 2 introduces the methodology of the proposed strategy, including an explanation of the used semantic segmentation networks and softmax regression. Section 3 presents training details of FCN-8s, SegNet, U-Net, PSPNet, and DeepLabv3+. Section 4 exposes ensemble learnings of the trained semantic segmentation networks using the softmax regression algorithm. Section 5 demonstrates results and discussions of concrete damage detection using the proposed strategy. Section 6 concludes this paper.

## 2. Methodology

**Figure 1:**
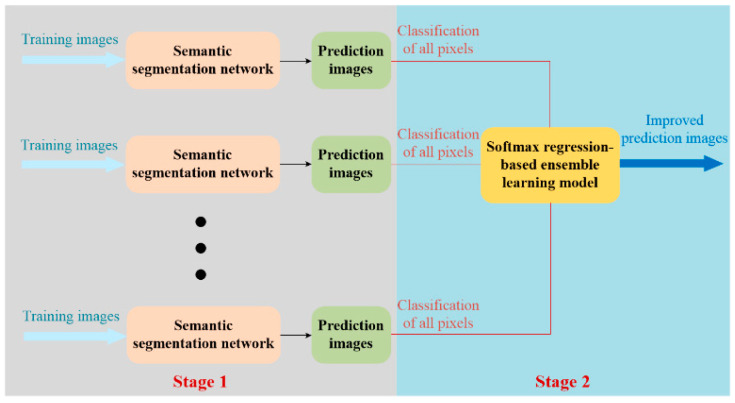
Concept diagram of performance improvement strategy for concrete damage detection using stacking ensemble learning of multiple semantic segmentation networks.

This section presents the methodology of this paper. Figure 1 shows a conceptual diagram of the performance improvement strategy for concrete damage detection using stacking ensemble learning across multiple semantic segmentation networks. The proposed performance improvement strategy includes two stages. In stage 1, images of concrete cracking and spalling are collected and labeled at the pixel level to build a database of concrete damage. Then semantic segmentation networks for stacking ensemble learning are trained using the built database, and then the types of damage in the images are predicted using the trained semantic segmentation networks. In stage 2, the classification of all the pixels in the prediction images from the trained semantic segmentation networks are extracted, and then the extracted pixel classification and their label are applied to the training softmax regression-based ensemble learning models. Finally, the trained softmax ensemble learning models will output the improved prediction images for damage detection.

In the proposed strategy, the semantic segmentation networks in stage 1 are named as the base models of ensemble learning. It is noted that at least two base models are required in an ensemble learning model, and any semantic segmentation network can be used as a base model in theory. The aim of this paper is to propose and prove that ensemble learning of multiple semantic segmentation networks can improve concrete damage detection performance. To realize this, we select five classical semantic segmentation networks (FCN-8s, SegNet, U-Net, PSPNet, and DeepLabv3+) as the base models of ensemble learning, although there are more models for semantic segmentation used in practice.

### 2.1. Semantic Segmentation Networks

Semantic segmentation networks, end-to-end and pixel-to-pixel convolutional networks, can classify and locate pixels in images. Encoder-decoder is a typical form for semantic segmentation networks. In this study, FCN-8s, SegNet, U-Net, PSPNet, and DeepLabv3+, five remarkable semantic segmentation networks, are employed to detect concrete cracks in images. After the five semantic segmentation networks are trained, they can individually complete concrete crack detection. To improve detection performance of concrete crack, ensemble learnings for the trained semantic segmentation networks are implemented.

#### 2.1.1. FCN-8s

A fully convolutional network (FCN) includes three models: FCN-32s, FCN-16s, and FCN-8s. FCN-8s is a skip structure where previous feature maps of pooling layers are fused to refine segmentation performance [38]. The FCN-8s can adapt to any size input because it does not include a full connection layer. Additionally, the skip structure of FCN-8s combines the feature maps of different depth layers, which ensures robustness and accuracy.

#### 2.1.2. SegNet

SegNet consists of an encoder and decoder [39]. The encoder is composed of the first 13 convolution layers and 5 pooling layers of the VGG (Visual Geometry Group) backbone with 16 convolution layers. In the decoder, feature maps are enlarged in up-sampling layers, where pooling indices recorded in previous pooling layers are adopted. The use of pooling indices saves the contour information of feature maps and reduces the number of network parameters.

#### 2.1.3. U-Net

The U-Net is composed of an encoder part and a symmetric decoder part of feature maps [40]. In the U-Net, the feature maps of the extension part are directly copied and cut to the same size as the upsampled image, and then they are connected to combine the features of different layers.

#### 2.1.4. PSPNet

In PSPNet, the first extract features of an input image using the pre-trained ResNet and the extracted feature maps are simultaneously passed through four parallel pooling layers for the respective upsampling across different scales in the pyramid pooling module [41]. Then the four groups of feature maps are fused with the extracted features of pre-trained ResNet, and a convolution layer is followed to predict the segmented output image. The pyramid pooling module in PSPNet can upsample feature maps in four scales, which improves segmentation information on a global scope.

#### 2.1.5. DeepLabv3+

Based on DeepLabv3 [45], DeepLabv3+ [42] is proposed. DeepLabv3+ adopts Xception as the backbone network and adds encoder and decoder modules. In the encoder and decoder modules, feature information of different sizes is fused. Atrous convolution is used to extract image features in the encoder module, which enlarges the receptive field without a loss of information. The application of the Xception network increases the robustness and running speed of semantic segmentation.

### 2.2. Softmax Regression-Based Ensemble Learning

Because limited images are used to train the semantic segmentation network model, a single network model often has insufficient performance in semantic segmentation network-based concrete damage detection methods. Ensemble learning can fuse multiple single semantic segmentation network models to enhance the overall damage detection performance. Ensemble learning is based on the theory that multiple weak classifiers can be equivalent to a strong classifier with high generalization and accuracy. Thus, ensemble learning of multiple semantic segmentation networks can solve the problems of small databases and local optimum to some extent, which single semantic segmentation networks cannot address. In ensemble learning, single semantic segmentation networks, which are also called base models, are diverse and differ from each other. Their outputs are complementary and can be used to improve the detection performance of concrete damage. In our study, the stacking method is used to fuse single semantic segmentation networks in ensemble learning processes, and the softmax regression algorithm is used to predict the final classification of damaged pixels in the images.

Softmax regression is a supervised learning algorithm, so both feature *x* and corresponding class label *y* are necessary when training the softmax regression model. In a given training set, {(*x_1_*, *y_1_*), (*x_2_*, *y_2_*), ···, (*x_m_*, *y_m_*)} of *m* examples where *y_i_* ∈ {0, *k*}, if *p*(*y = j|x_j_*) is the predictive probability for class *j* where the class label with a higher probability is output as the classification result, hypothesis function of softmax regression can be defined as follows.

(1) $$h_{w}(x_{i})=\left[\begin{array}{c} p(y_{i}=1|x_{i};w)\\ p(y_{i}=2|x_{i};w)\\ \cdot \cdot \cdot \\ p(y_{i}=k|x_{i};w) \end{array}\right]=\frac{1}{\sum_{j=1}^{k}e^{w_{j}^{T}x_{i}}}\left[\begin{array}{c} e^{w_{1}^{T}x_{i}}\\ e^{w_{2}^{T}x_{i}}\\ \cdot \cdot \cdot \\ e^{w_{k}^{T}x_{i}} \end{array}\right]$$

where $w_{1}^{T},\;w_{2}^{T},\;\cdots ,\;w_{k}^{T}\in R^{n+1}$ are the weights of the softmax regression model, and $1/\sum_{j=1}^{k}e^{W_{j}^{T}x_{i}}$ normalizes the distribution so that all predictive probability sums to one.

Cost function used by softmax regression can be written according to the log-likelihood in Equation (2).

(2) $$J(w)=-\frac{1}{m}\left[\sum_{i=1}^{m}(1-y_{i})\log(1-h_{w}(x_{i}))+\sum_{i=1}^{m}y_{i}\log h_{w}(x^{i})\right]$$

According to Equation (1), the cost function can also be written as Equation (3).

(3) $$J(w)=-\frac{1}{m}\left[\sum_{i=1}^{m}\sum_{j=1}^{k}1\left\{y_{i}=j\right\}\log\frac{e^{w_{j}^{T}x_{i}}}{\sum_{l=1}^{k}e^{w_{l}^{T}x_{i}}}\right]$$

where 1{·} is an indicator function, such that 1{a true statement} = 1 and 1{a false statement} = 0.

By adding a weight decay term to the Equation (3), the cost function takes the form as follows.

(4) $$J(w)=-\frac{1}{m}\left[\sum_{i=1}^{m}\sum_{j=1}^{k}1\left\{y_{i}=j\right\}\log\frac{e^{w_{j}^{T}x_{i}}}{\sum_{l=1}^{k}e^{w_{l}^{T}x_{i}}}\right]+\frac{\lambda }{2}{\sum_{i=1}^{k}\sum_{j=0}^{n}w_{ij}}^{2}$$

In the training process of softmax regression, the goal is to minimize the cost function where the parameters of weight *w* are the optimal parameters for given training examples. Many optimization algorithms, such as gradient descent and L-BFGS, are guaranteed to converge to the global minimum on the basis of Equation (4). In our study, gradient descent is applied to find the gradient variation and cost function minimum.

## 3. Building Semantic Segmentation Database of Concrete Damage

**Figure 2:**
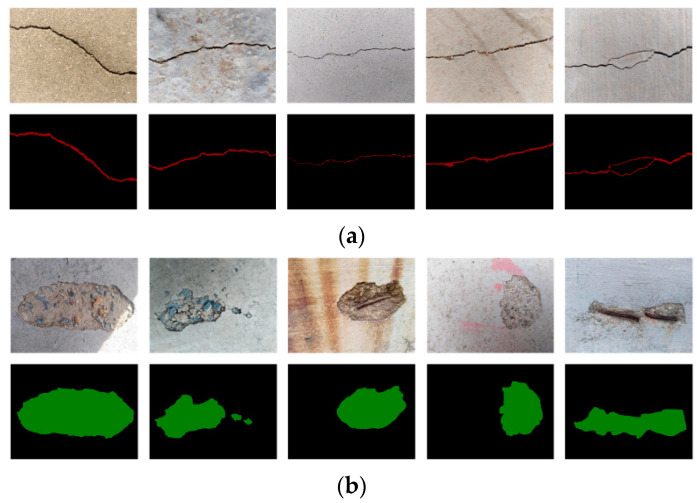
Examples of damage images and their labels in built database: (**a**) crack and (**b**) spalling.

To train the FCN-8s, SegNet, U-Net, PSPNet, and DeepLabv3+, we build a semantic segmentation database including 500 concrete crack and spalling images with 4032 × 3046 pixel resolution. These images come from the concrete damage database built in our previous study [32,46]. They are taken from concrete grounds, walls, bridges, and other concrete structures. To decrease computational cost of training semantic segmentation networks, the 500 raw images and their labels are resized into smaller images of 504 × 376 pixel resolution. Figure 2 shows some examples of the damage images and their labels in the built database.

According to the fivefold cross-validation principle, 400 images are randomly chosen to train the semantic segmentation network base models from the 500 images, and the remaining 100 images are used as a testing set.

## 4. Stacking Ensemble Learning-Based Concrete Damage Detection

In this section, FCN-8s, SegNet, U-Net, PSPNet, and DeepLabv3+ are trained as base models of ensemble learning. Then ensemble learning is carried out according to the prediction of the trained base models to improve detection performance. All the described work is based on Pytorch in a Linux operating system and performed on a workstation that is configured with a graphic processing unit (GPU) (CPU: Intel(R) Core(TM) i9-10900X @ 3.70 GHz, RAM: 64 GB, GPU: NVIDIA GeForce RTX 3090).

### 4.1. Training of Semantic Segmentation Network Base Models

**Figure 3:**
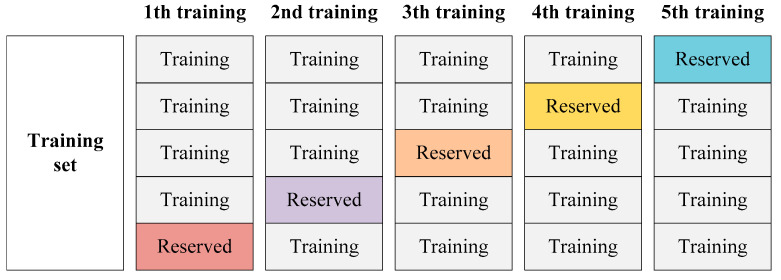
Training data of each semantic segmentation network base model.

When implementing training for the semantic segmentation network base models, we divide the built training set into five parts for the next cross-validation of the base model training. Figure 3 presents the training data of each semantic segmentation network base model. In the training processes of base models, FCN-8s, SegNet, U-Net, PSPNet, and DeepLabv3+ are trained five times on four parts of the divided training set, respectively, and the one remaining part of the divided training set is reserved. The five reserved parts of the training set will be predicted as the training data of the following ensemble learning. The purpose of doing this is to avoid overfitting the trained base model. To make use of the collected images and decrease the probability of overfitting, we used the data augmentation methods of random cropping, flipping, and rotation on the training data.

The FCN-8s, SegNet, U-Net, PSPNet, and DeepLabv3+ are trained using a batch stochastic gradient descent optimizer with 16 images inputted in each batch and a total of 100 training epochs (2000 iterations) are implemented. The annealing learning rate over training iterations is helpful in achieving a good network model. Therefore, the effective learning rate of the semantic segmentation networks follows a polynomial decay to reduce the initial learning rate to be zero in FCN-8s, U-Net, PSPNet, and DeepLabv3+. The rest of the training parameters for the five semantic segmentation networks are listed in Table 1, where the network backbone represents the convolutional neural network used to extract damage features in each semantic segmentation network. It should be noted that all the parameters in Table 1 have been optimized in our study.

**Figure 4:**
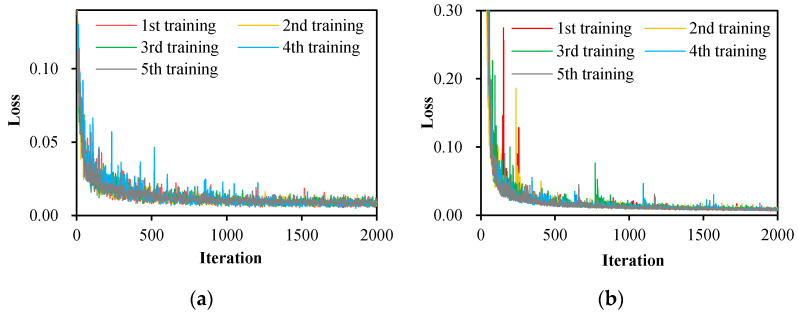

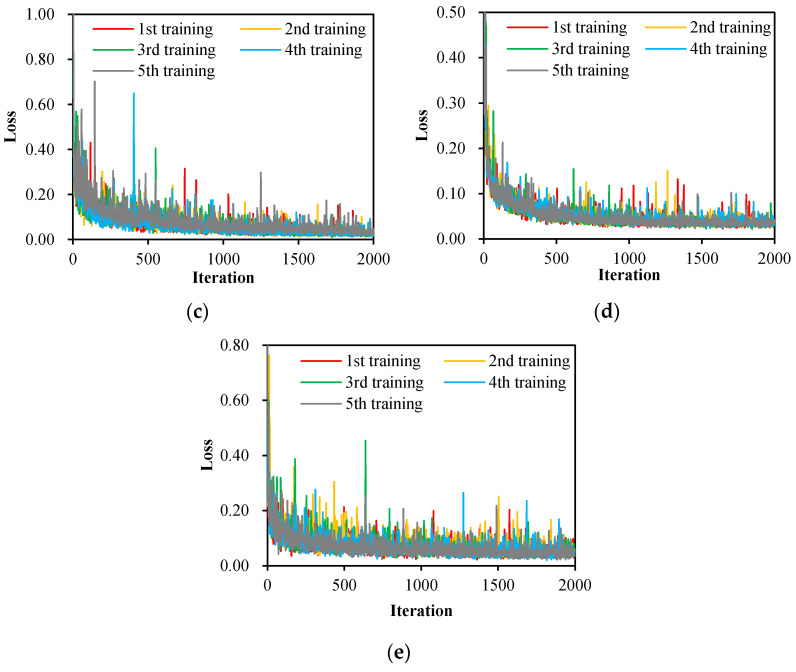
Training losses over iterations of five semantic segmentation networks: (**a**) FCN-8s, (**b**) SegNet, (**c**) U-Net, (**d**) PSPNet, and (**e**) DeepLabv3+.

Using the hyperparameters in Table 1, the FCN-8s, SegNet, U-Net, PSPNet, and DeepLabv3+ are trained five times according to the training data presented in Figure 3. With a GPU boosting the training processes, the recorded training time for each semantic segmentation network is 21–30 min. Figure 4 shows recorded training losses over iterations of the five networks. It can be observed that all the losses decreased quickly in the early training and converged to less than 0.05 in the end.

### 4.2. Softmax Regression-Based Ensemble Learning of Semantic Segmentation Networks

#### 4.2.1. Training and Testing Data of Ensemble Learnings

**Figure 5:**
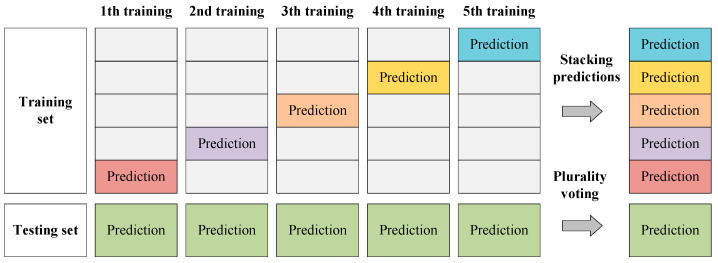
Generating process of training and testing data for ensemble learning in each network.

Following Section 4.1, the predictions of the trained FCN-8s, SegNet, U-Net, PSPNet, and DeepLabv3+ are implemented on the testing set, and one part of the divided training set is reserved. The prediction time for each semantic segmentation network is recorded as 1 s for each image. Figure 5 shows the generative process of training and testing data for the softmax regression-based ensemble learning in each network. In the process, the classifications of all pixels in the prediction results are extracted, and the extracted pixel classifications in predictions of the one reserved part of the divided training set are stacked as the training data of ensemble learning. The extracted pixel classes for the predictions testing set are voted for as the testing data for ensemble learning according to plurality voting.

**Figure 6:**
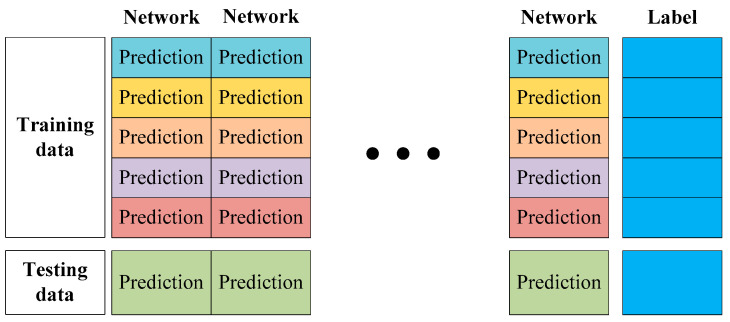
Composition of generated training and testing data for ensemble learning.

The training and testing data for ensemble learning are composed of pixel classifications for predictions from single semantic segmentation networks. For the training and testing data, their true classes as labels are extracted from the built image database. The composition of the generated training and testing data for ensemble learning are presented in Figure 6. The classifications of all pixels in prediction images of semantic segmentation network base models and their labels are applied as training data. The pixel classifications from plurality voting for the testing images are used as the testing data.

#### 4.2.2. Training and Testing Ensemble Learning Models

In our study, four ensemble learning models are generated using the trained semantic segmentation network base models in Section 4.1. In the first ensemble learning model, the FCN-8s and SegNet are used as base models. Incrementally, ensemble learning is carried out using FCN-8s, SegNet, and U-Net in the second model. In the third ensemble learning model, FCN-8s, SegNet, U-Net, and PSPNet are applied as base models. In the last model, ensemble learning is implemented using all the trained five semantic segmentation network base models.

**Figure 7:**
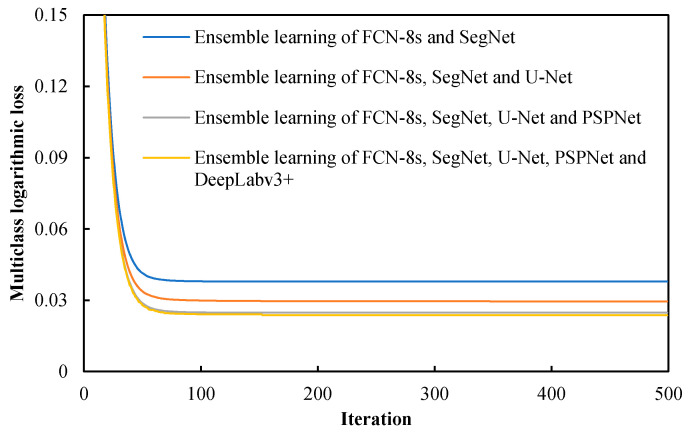
Multiclass logarithmic losses over iterations of four ensemble learning models.

The four ensemble learning models are trained using 500 iterations with a learning rate of 0.1, a max depth of three, and a *λ* in Equation (4) of 0.8. When training ensemble learning models, a multiclass logarithmic loss is recorded in every iteration. Figure 7 represents the recorded multiclass logarithmic losses over iterations of four ensemble learning models. It can be observed that the loss decreased quickly in the first 100 iterations. Additionally, there is a clear trend that the more the number of base models, the lower the convergence losses achieved by ensemble learning models.

**Figure 8:**
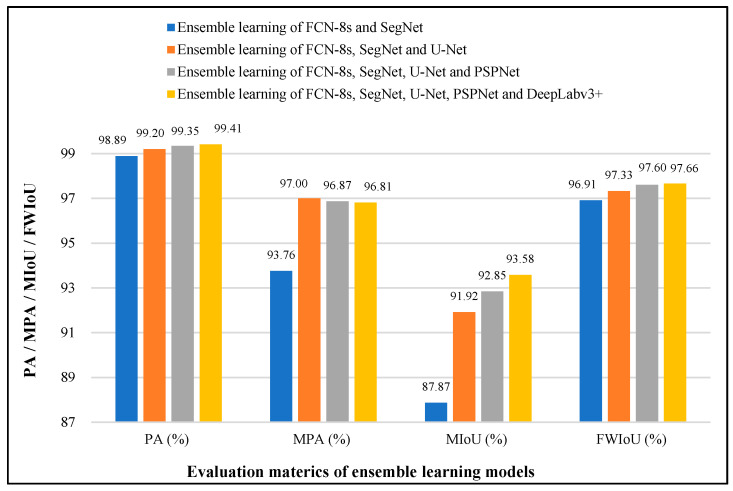
The testing performance of four ensemble learning models.

After the training of ensemble learning models, testing is implemented using the testing data presented in Figure 6 to validate the performance of the trained models. In the testing processes, the pixel accuracy (PA), mean pixel accuracy (MPA), mean intersection over union (MIoU), and frequency-weighted intersection over union (FWIoU) are used as evaluation metrics for the detection performance of concrete damage. The testing performance of four ensemble learning models is shown in Figure 8. It can be seen in Figure 8 that the PA, MIoU, and FWIoU of four ensemble learning models achieve continued growth with the increasing number of base models, especially MIoU. The MIoU of the first ensemble learning model of FCN-8s and SegNet is 87.87%. After fusing the U-Net, the second ensemble learning model has a 4.05% improvement over the first one in MIoU. The third and fourth models provide 0.93% and 0.73% improvement over previous models, respectively. Apart from the PA, MIoU, and FWIoU, the MPA for the ensemble learning models, FCN-8s, SegNet, and U-Net, provide a greater increase, but the ensemble learning models which use more base models do not further improve in this category.

**Figure 9:**
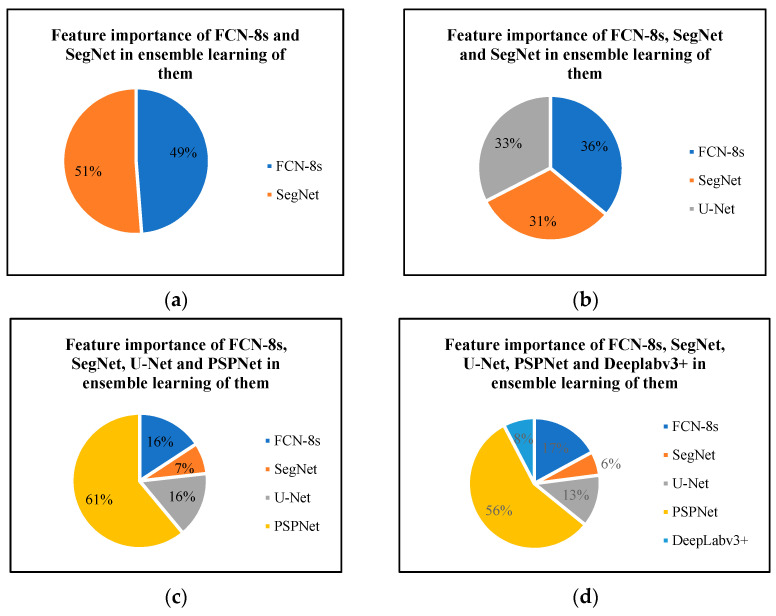
Feature importance of semantic segmentation network base models in softmax regression-based ensemble learnings: (**a**) ensemble learning of FCN-8s and SegNet, (**b**) ensemble learning of FCN-8s, SegNet, and U-Net, (**c**) ensemble learning of FCN-8s, SegNet, U-Net, and PSPNet and (**d**) ensemble learning of FCN-8s, SegNet, U-Net, PSPNet, and DeepLabv3+.

When training the ensemble learning models, the pixel classifications of prediction results from the semantic segmentation network base models are used as features, where the features and the corresponding label make up learning samples for ensemble learning model training. However, the importance of each feature from each semantic segmentation network base model is different. Figure 9 presents the feature importance of each semantic segmentation network in four ensemble learning models, where the importance is computed according to the normalized average gain across all splits where the feature is used. We can find that the importance of base models in the first two ensemble learning models are nearly equal, but the PSPNet base model shows the highest feature importance in the latter two ensemble learning models, which indicates that the PSPNet makes the greatest contribution to damage pixel classification in the latter two ensemble learning models.

## 5. Results and Discussions

**Figure 10:**
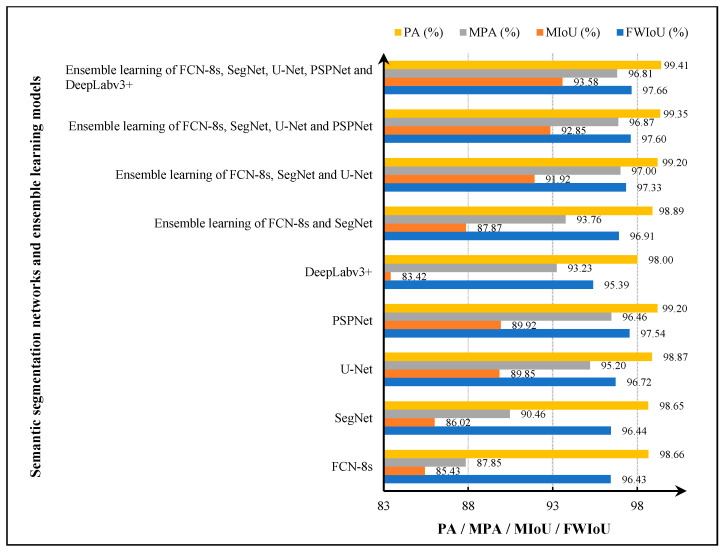
Performance comparison of five semantic segmentation networks and four ensemble learning models.

To validate the performance improvement of concrete damage detection using the proposed stacking ensemble learning-based strategy, the FCN-8s, SegNet, U-Net, PSPNet, and DeepLabv3+ are retrained and retested using the 400 training images and the 100 testing images from the built database. Then the testing results of the five semantic segmentation networks and four ensemble learning models are compared, and their performance comparison is presented in Figure 10. It can be seen in Figure 10 that the softmax regression-based ensemble learning of FCN-8s, SegNet, U-Net, PSPNet, and DeepLabv3+ achieves the best PA of 99.41%, MIoU of 93.58%, and 97.66% of FWIoU, which are 0.21%, 3.66%, and 0.12% higher than the best single semantic segmentation network PSPNet. Moreover, the ensemble learning of FCN-8s, SegNet, and U-Net provides the best MPA of 97%, which is also 0.54% higher than the PSPNet.

Compared with single semantic segmentation networks, the proposed stacking ensemble learning-based strategy can improve the detection performance of concrete damage effectively. Additionally, to a certain extent, the detection performance can be sustainably improved by adding a number of semantic segmentation network base models. It is meaningful for the application scenario where detection accuracy is desired, but the accuracy of a single semantic segmentation network is insufficient. In addition, the performance of a single semantic segmentation network base model is important for ensemble learning. A better base model will make a greater contribution and show more feature importance in the ensemble model. Therefore, training high-performance semantic segmentation network base models is helpful in implementing following ensemble learning.

In ensemble learning, plurality voting is a typical way of fusing base models. To compare the damage detection performance of plurality voting and softmax regression-based fusion methods, we fuse the FCN-8s, SegNet, U-Net, PSPNet, and DeepLabv3+ using the plurality voting-based ensemble learning method. The plurality voting-based ensemble learning model shows 99.19% PA, 95.75% MPA, 91.65% MIoU, and 97.29% FWIoU, which are 0.22%, 1.06%, 1.93%, and 0.37% lower than the PA, MPA, MIoU, and FWIoU of the softmax regression-based ensemble learning model. The comparison results indicate that the proposed softmax regression-based ensemble learning method can provide an effective performance improvement in concrete damage detection.

## 6. Summary and Conclusions

This paper proposed a performance improvement strategy for concrete damage detection using stacking ensemble learning of multiple semantic segmentation networks. The semantic segmentation networks were applied to detect coarse concrete crack and spalling, and then ensemble learning models were used to improve the detection results. A database including 500 crack and spalling images with 504 × 376 pixel resolutions was built. The FCN-8s, SegNet, U-Net, PSPNet, and DeepLabv3+ were trained using a four-fifth training set, and the predicted images from the reserved one-fifth of the training set from the trained semantic segmentation networks were stacked. The classifications of all pixels in the stacked prediction images and their labels make up training data for ensemble learnings. Then four softmax regression-based ensemble learning models were trained using the training data. To validate the performance improvement of concrete damage, the FCN-8s, SegNet, U-Net, PSPNet, and DeepLabv3+ were respectively trained using all the images in the training set, and the damage detection performance comparison for the testing sets was studied between the five semantic segmentation networks and the four ensemble learning models. The comparative study showed that the PA, MPA, MIoU, and FWIoU could be improved by 0.21%, 0.54%, 3.66%, and 0.12%, respectively.

The proposed stacking ensemble learning-based strategy can improve the detection performance of concrete damage effectively. The performance of a single semantic segmentation network base model is important for ensemble learning, where a better base model will make a greater contribution and show more feature importance in the ensemble learning model. Moreover, to a certain extent, the detection performance of concrete damage can be sustainably increased by adding a number of semantic segmentation network base models. The proposed strategy can also be applied to the ensemble learnings of convolutional neural networks, object recognition networks, and instance segmentation networks to improve the network performance and cope with the application scenario, where high accuracy is required, but the accuracy of a single semantic segmentation network is insufficient.

The proposed method is strong at detecting concrete damages in the original database. Because deep learning models have to learn damage features from much training data, one common shortcoming of almost all deep learning-based detection approaches is the inability to detect concrete damage in images that are not included in the original database. To increase the detection performance of the proposed method, in future studies, more images with more types of concrete damage under various conditions will be collected and added to the existing database.

## Figures and Tables

**Table 1:** Training Hyperparameters for Five Semantic Segmentation Networks.

	Networks	FCN-8s	SegNet	U-Net	PSPNet	DeepLab v3+
Parameters						
Backbone		VGG-16	VGG-16	-	ResNet-50	Xception
Initial learning rate		0.1	0.01	0.01	0.01	0.1
Weight decay		0.0001	0.0005	0.0001	0.0001	0.0001
Momentum		0.9	0.9	0.99	0.9	0.9
Loss function		Cross entropy	Weighted softmax	Cross entropy	Cross entropy	Cross entropy

Note: ‘-’ represents when no existing convolution neural network is used as network backbone.

## Data Availability

The data presented in this study are available upon request from the corresponding author.
